# Cholera Infantum

**Published:** 1884-09

**Authors:** John V. Allen

**Affiliations:** Philadelphia


### CHOLERA INFANTUM.

JOHN V. ALLEN, M. D., PHILADELPHIA.

The summer season on which we have entered presented to us numerous cases of all stages of this disease, of which a vast majority will prove fatal if we do not exercise our keenest judgment in selecting diet and the proper homœopathic remedy.

This disease attacks children of every age, but by far most common in the first and second year, at which period it exercises its most destructive influence.

This very fact shows that the manner of nourishment, especially the artificial, or the transition from the breast to weaning, plays an important part, especially as infants who receive good mother's or nurse's milk are much more rarely affected than others.

Every physician knows that with the first warm days of early summer cases of this disease make their appearance, increase in frequency every week until they become epidemic, and finally disappear gradually in September, although some "feelers" of the epidemic are observed in October.

The nature of the disease is, however, entirely unknown. Definite forms of bacteria to which the infectious character can be attributed have not been found. But we may hope for more satisfactory results from further investigation if we take into consideration the cases of intestinal mykosis reported in literature.

I will narrate but a few of the symptoms of this disease. In the early stage or beginning there are rapidly following brownish yellow or greenish, thin evacuations; pain is absent or very mild; apart from anorexia and increased thirst the general condition may be unaffected.

In other series of cases the disease begins with more violent symptoms. Profuse watery evacuations and vomiting follow one another rapidly. The severity of the latter varies. At times it is rare, then very frequent, occurring whenever fluids are ingested. The rapid effect on the vital force is common to all, and occurs so much more rapidly the younger the child, though it is not absent in older children, and, as you know, even in adults. Great weakness, pallor of the skin, sinking in of the eyes in the orbits, coldness of the cheeks, hands, and feet, increasing frequency and smallness of the pulse, feeble voice, slight cyanosis of the face and mucous membrane indicate the depression of the power of the heart. The primary restlessness and jactitation soon pass into an apathetic, somnolent condition.

The treatment of this disease you all know, and I therefore will take up no more valuable time, as each case must be individualized, and any one of a hundred or more remedies may be indicated or called for.

I will mention for your interest one case of the present season.

Baby S., age, seven weeks; called on May 10th, 1884. Child had been sick four days under allopathic care, and rapidly sinking. I found the child in a collapsed condition; surface of body cold, especially extremities; eyes sunken and blueness around the lids and lips; anterior fontanell sunken; violent vomiting with straining, and stools green and watery. I gave four powders of Ipecac.$^{5cm}$ to be followed by Sac. lac., and saw patient eight hours later; mother reported no vomiting since taking the first dry powder; the child's face presented better color; blueness of lips and eyelids gone, and surface of body much warmer, but has had two evacuations, green, as before; continued Sac. lac.

Next day's report: Patient has had four passages of same color, but of a sour smell, and the mother said the "baby sweat a great deal about the head, wetting the pillow;" gave patient two powders of Calc. os.$^{cm}$, and on following day the mother reported one passage within the last twenty-four hours, which was of a natural color and consistency. The child continued to rapidly improve from this on under Sac. lac., and is now entirely well.

